# Copy number variation analysis in the context of electronic medical records and large-scale genomics consortium efforts

**DOI:** 10.3389/fgene.2014.00051

**Published:** 2014-03-18

**Authors:** John J. Connolly, Joseph T. Glessner, Berta Almoguera, David R. Crosslin, Gail P. Jarvik, Patrick M. Sleiman, Hakon Hakonarson

**Affiliations:** ^1^The Center for Applied Genomics, Children’s Hospital of PhiladelphiaPhiladelphia, PA, USA; ^2^Department of Pediatrics, University of Pennsylvania Perelman School of MedicinePhiladelphia, PA, USA; ^3^Departments of Medicine (Medical Genetics) and Genome Sciences, University of Washington Medical CenterSeattle, WA, USA

**Keywords:** CNV, copy number, structural variation, eMERGE, review

## Abstract

The goal of this paper is to review recent research on copy number variations (CNVs) and their association with complex and rare diseases. In the latter part of this paper, we focus on how large biorepositories such as the electronic medical record and genomics (eMERGE) consortium may be best leveraged to systematically mine for potentially pathogenic CNVs, and we end with a discussion of how such variants might be reported back for inclusion in electronic medical records as part of medical history.

## WHAT ARE COPY NUMBER VARIATIONS?

Copy number variations (CNVs) are deletions and duplications in the genome that vary in length from ~50 base pairs to many megabases (50 base pair to 1 kilobase CNVs are typically considered indels). Events that cause CNVs include non-allelic homologous recombination, non-homologous end-joining, transposition of transposable elements, transposition of pseudogenes, variable numbers of tandem repeats, and replication errors following template-switching or fork stalling. CNVs are the primary mode by which an individual acquires a mutation, and occur at a rate of approximately 1.7 × 10^-^^6^ per locus as opposed to 1.8 × 10^-^^8^ for sequence variation (Lupski, 2007). Estimates of CNV frequency vary depending on the size of the structural variation classed as CNV – some estimates suggest that up to 12% of the genome may be variable in copy number, and that the cumulative result of CNV inheritance may constitute more than 10% of the human genome (Carter, 2007; Lupski et al., 2010). Recent studies suggest that the average human genome contains >1000 CNVs, covering approximately four million base pairs (Conrad et al., 2010; Mills et al., 2011), and occur at a rate of 0.07–0.12 per generation (Cordaux and Batzer, 2009; Itsara et al., 2010; Beck et al., 2011; Malhotra and Sebat, 2012). The Database of Genomic Variation (DGV)^1^ currently lists over 100,000 published, unique, CNVs across the genome. While the majority continues to be benign, an increasing number of CNVs have been associated with disease susceptibility. Common functional consequences of CNVs typically demonstrate gene dose effect and include truncated protein sequences, eliminated/reduced protein expression (typically the result of deletions), or increased protein expression (typically caused by duplications).

## HOW ARE COPY NUMBER VARIATIONS IDENTIFIED?

### ARRAY-BASED APPROACHES

**FIGURE 1:**
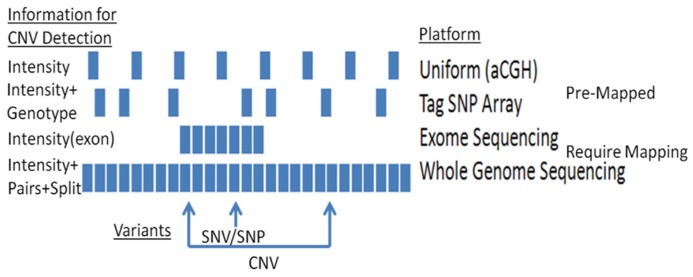
**CNV detection using different platforms: platforms vary in their capacities to detect CNVs**.

**FIGURE 2:**
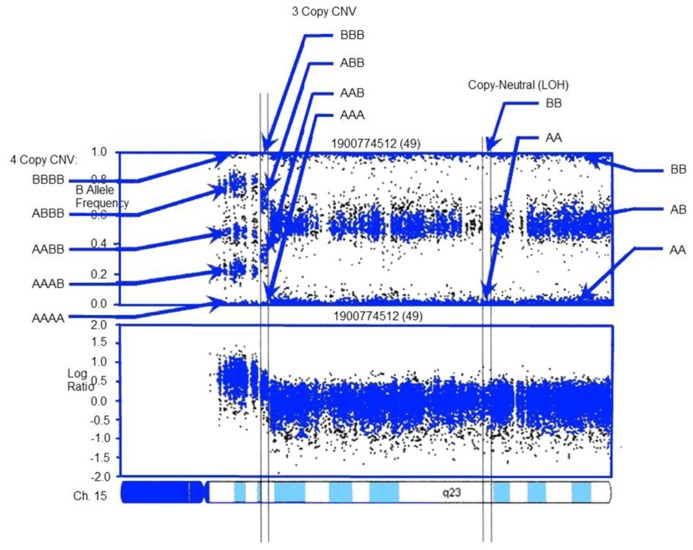
**CNV detection in SNP-array data using PennCNV: example log R ratio (LRR) and B Allele Freq (BAF) values for the chromosome 15 q-arm of an individual**. Three normal chromosomal BAF genotype clusters (AA, AB, and BB genotypes) have LRR values around zero. The copy-neutral loss-of-heterozygosity (LOH) region has normal LRR values, but no AB cluster. Increased copy number can be observed in the increased number of peaks in the BAF distribution and increased LRR values. LRR and BAF patterns are different for different CNV regions, and can be used to generate CNV calls. Adapted from Wang et al. (2007).

A range of approaches are available for detecting CNVs (**Figure 1**). The most common methods rely on computational methods, which leverage signals from genotyping and sequencing to infer CNVs. For example, large chromosomal anomalies can be detected through log R ratio (LRR) and B-allele frequency (BAF), data routinely generated and provided with single nucleotide polymorphism (SNP) and exome microarrays (e.g., **Figure 2**). For replication and validation, quantitative PCR – which compares the threshold cycles of a target versus reference sequence – is still widely deployed. In a similar vein, paralogs-ratio testing and molecular copy number counting are also used for validation.

For high-throughput CNV detection, the most common platforms are genome hybridization (CGH) arrays, genome-wide association (GWA) arrays, and second-generation sequencing (SGS). CGH arrays use artificial bacterial chromosomes or long synthetic oligonucleotides to probe either specific regions of interest or the entire genome (Greshock et al., 2007; Haraksingh et al., 2011).While this method has relatively lowspatial resolution (typically >5–10 Mb; Kallioniemi et al., 1993) and requires a relatively large volume of DNA, CGH does offer high sensitivity and specificity (Greshock et al., 2007; Haraksingh et al., 2011), which is critical in a diagnostic context.

Single nucleotide polymorphism (SNP) arrays are more commonly used for CNV analysis, and CNVs can be identified from standard GWA array signals, or from arrays that utilize custom probes. Custom probes offer greater coverage of non-SNP sites, and can offer high sensitivity, particularly with regard to breakpoint resolution (Haraksingh et al., 2011). While conventional (i.e., non-custom) SNP arrays offer less specificity, they nevertheless represent a cost-effective option for characterizing CNVs and have been successfully applied to a wide range of phenotypes to date (Connolly and Hakonarson, 2012).

Importantly, it is possible to retroactively characterize CNVs from existing genome-wide association study (GWAS) data. In this context, the observed SNP signal of an allele relative to the normalized intensity of the allele can be used to deduce a deletion (decreased intensity) or duplication (increased intensity; Glessner et al., 2012). This possibility constitutes a major opportunity for custodians of large biorepositories such as electronic medical record and genomics (eMERGE), where a large volume of GWAS data has already been generated. Since its founding in 2007, the eMERGE consortium has produced dozens of GWASs on a range of phenotypes including lipids (Rasmussen- Torvik et al., 2012), arrhythmia (Ritchie et al., 2013), and white blood cell count (Crosslin et al., 2012) to name a few. For many of these phenotypes, no CNV studies have been published to date. This, we believe, represents an opportunity to identify new disease-associated loci without the generation of new genotype data, and will be addressed by the consortium in the immediate future. Similarly, we note that a large number of studies listed in the NHGRI GWAS catalog^2^ do not have complementary CNV data, suggesting a largely under-utilized resource.

For array-based analyses, a range of packages are available. Both Affymetrix and Illumina – the two primary purveyors of SNP arrays – offer free software packages for CNV analysis. Independently developed toolsets are also available. These include circular binding segmentation (Olshen et al., 2004) MixHMM (Liu et al., 2010), GADA (Pique-Regi et al., 2008), PennCNV (**Figure 2**; Wang et al., 2007), and ParseCNV (Glessner et al., 2013a; the latter two were developed by eMERGE researchers and are widely used).

### SEQUENCING-BASED APPROACHES

Common CNVs are well-covered by SNPs in existing arrays (Conrad et al., 2010; Wellcome Trust Consortium et al., 2010). However, a resequencing study by Pang et al. (2010) suggests that coverage of rare CNVs may be less comprehensive. The authors identified over 12,000 structural variants in 4,867 genes across 40 + mb of sequence (the Venter genome), which had been initially unreported. More than 24% of these CNVs would not have been imputed by SNP-association. Given that rare alleles can have large effect sizes and a high penetrance, these results underline the limitations of SNP arrays to identify certain pathogenic CNVs. SGS, which is far more proficient at identifying rare CNVs, offers an attractive solution in this regard – particularly in identifying novel insertions absent in the reference genome. This has obvious clinical utility. SGS also confers a number of other critical advantages in terms of ability to identify smaller CNVs (<50 bp), and an enhanced capability for detecting breakpoints (Li and Olivier, 2013). Indeed, because SGS allows us to probe breakpoints at the level of base pairs, it facilitates capture of the signature of potential mutational mechanisms (Li and Olivier, 2013).

With SGS data, the most common methods for CNV identification from short-read analysis (Medvedev et al., 2010) are read-depth analysis (Xie and Tammi, 2009; Yoon et al., 2009; Abyzov et al., 2011), split-read mapping (Mills et al., 2006), paired-end read mapping (Korbel et al., 2009), and clone-based sequencing (Kidd et al., 2008). For all approaches, the most important determinants of accuracy are alignment and read-length. The average length of (reliable) reads is ~ from 100 to 150 bp, which is insufficient to eliminate erroneous mapping. As this metric improves, CNV-calling algorithms will become more accurate.

A large number of algorithms have been developed for indentifying CNVs from sequencing data, including CNVnator (Abyzov et al., 2011), PennCNV-Seq (in press), GenomeStrip (Handsaker et al., 2011), cnvHiTSeq (Bellos et al., 2012), and XHMM (Fromer et al., 2012). Different CNV algorithms have different strengths and weaknesses (see Li and Olivier, 2013 for review), and the most effective strategy in terms of minimizing erroneous CNV calls is to incorporate multiple toolsets, which can be validated computationally via local *de novo* assembly (e.g., see SVMerge, Wong et al., 2010).

## DISEASE-ASSOCIATED COPY NUMBER VARIATIONS

As discussed elsewhere in this issue, GWASs have been successful in identifying common risk variants, particularly where the frequency of such variants is >5%. In addition to common variants, certain disorders have been shown to be enriched for rare CNVs (Conrad et al., 2010; Pang et al., 2010). In terms of functional impact, CNVs have been shown to be enriched in genes involved in immune responses, cell–cell signaling, and retrovirus- and transposition-related protein coding (Li and Olivier, 2013). A large number of phenotypes have now been associated with CNVs, including several rare diseases (Matsuura et al., 1997) and a range of neurodevelopmental disorders (Glessner et al., 2012), including depression (Glessner et al., 2010c), schizophrenia (Glessner et al., 2010b), and autism (Glessner et al., 2009). Autism provides a particularly good example of how our understanding of genetic risk factors and etiology is enhanced by CNV research, as demonstrated by a recent exome sequencing study (Iossifov et al., 2012) involving 343 families from the Simons Simplex Collection.

The study identified 59 “likely gene disruptions (LGDs)” in autism cases. Interestingly, the 59-strong LGD shared overlapped strongly with a set of 842 proteins that interact with the fragile X protein, FMRP. In total, 14 of the 59 LGDs encoded FMRP-interacting proteins (*P* = 0.006), as did 13 of 72 CNV candidates from the group’s previous CNV paper (*P* = 0.0004). Thus, 26 of 129 candidates were FMRP-related (*P* < 1 × 10^-^^13^). These results mark the fragile X mental retardation 1 (*FMR1*) gene as a high-profile autism candidate. Screening upstream targets of *FMR1*, the same group identified a deletion in *GRM5* that removes a single amino acid, causing an additional substitution at the same site. *GRM5* encodes the glutamate receptor mGluR5 (Bear et al., 2004), which has been proposed as translational target in both ASD and ADHD (Elia et al., 2012; Silverman et al., 2012).

Several other CNV studies of autism have uncovered rare recurrent CNVs that have been informative. Our laboratory recently identified a range of CNVs in two major gene networks, ubiquitins and neuronal cell adhesion molecules that predispose to autism (Glessner et al., 2009). The ubiquitin–proteasome system is known to operate at pre- and post-synapses, and mediate neurotransmitter release, recycling of synaptic vesicles in pre-synaptic terminals, and modulating changes in dendritic spines and post-synaptic density (Yi and Ehlers, 2005). Neuronal cell adhesion molecules contribute to neurodevelopment by facilitating axon guidance, synapse formation and plasticity, and neuron–glial interactions.

Results from these and several other CNV studies suggest that genomic hotspots may be particularly vulnerable, which for autism include loci on chromosomes 1q21, 3p26, 15q11–q13, 16p11, and 22q11 (Bucan et al., 2009; Glessner et al., 2009; Pinto et al., 2010). Interestingly, these hotspots are part of large gene networks that are important to neural signaling and neurodevelopment, and have additionally been associated with other neuropsychiatric disorders. For example, studies of schizophrenia have highlighted structural mutations incorporating chromosomes 1q21, 15q13, and 22q11 (Glessner et al., 2010b). From an etiological perspective, autism and schizophrenia seem extremely different and it would seem counter-intuitive that associated loci should overlap. Some authors have addressed this peculiarity by proposing that the two disorders may in fact be opposite poles of the same spectrum (Crespi and Badcock, 2008). While such propositions await confirmation, they do highlight the potential of CNV studies to generate new hypotheses about the nature of complex diseases. Although individual structural variants explain relatively little by way of genetic variance, their cumulative is likely to be considerable. For autism, Marshall et al. (2008) suggested that CNVs play a causal role in 7% cases.

Beyond neuropsychiatric diseases, CNV studies have been published across a range of disease types, including heart disease (Goldmuntz et al., 2011), obesity (Glessner et al., 2010a), and cancer (Kuusisto et al., 2013). They have also recently been implicated in altered lifespan through alternative splicing mechanism (Glessner et al., 2013b).

## COPY NUMBER VARIATIONS IN THE CONTEXT OF THE EMERGE CONSORTIUM

As illustrated in **Table 1**, the eMERGE consortium biorepository includes ~60,000 individuals that have been genotyped on high-density GWA arrays^3^, all of which have been linked with electronic medical records (EMRs). The size and diversity of the repository is such that it invokes the possibility for deep mining of disease-associated variants across multiple phenotypes. It is inevitable that a reasonable proportion of these individuals have disease-associated CNVs, and a larger proportion may be carriers of structural variants in recessive disease genes. By systematically characterizing CNVs across the biorepository, we have a very obvious opportunity to catalog CNVs and their disease-burden status. We have now run PennCNV on eMERGE Phase I data (2007–2011), and will soon have circular binary segmentation analyses complete for the same set (50-kb to whole-chromosome). Relevant analyses will play a major role in the consortium’s Phase II genomics program (2012–2015).

**Table 1:** Summary of biorepositories and electronic medical records (EMRs) at 10 eMERGE-Institutions. Adapted from Gottesman et al. (2013).

Institution	Biorepository	Recruitment model	Biorepository size	Race/ethnicity and age of donors
Boston Children’s Hospital	Gene Partnership	Outpatient and hospital-based	3,372	83% European 9% African 6% Asian 11% Hispanic/Latino Mean age: 23 years
Children’s Hospital of Philadelphia	A Study of the Genetic Causes of Complex Pediatric Disorders	Population-based and disease-specific	60,000 internal (plus 100,000 external)	47.0% European 43.3% African 7.0% Admixed 1.7% Asian 0.8% Hispanic 0.2% Native Amer. Mean age: 11 years
Cincinnati Children’s Hospital	Better Outcomes for Children	Outpatient and hospital-based	8,472	73% European 10% African Mean age: 9 years
Geisinger Clinic	MyCode^®^	Population-based and disease-specific	35,000	98% European Age: < 89 years
Group Health Seattle	ACT Study; Alzheimer’s Disease Patient Registry (ADPR); Northwest Institute of Genetic Medicine (NWIGM)	Disease-specific and HMO-based	5,859	92% European Age: > 50 years
Marshfield Clinic Research Foundation	Personalized Medicine Research Project	Population-based	20,000	98% European Mean age: 48 years
Mayo Clinic	Vascular disease biorepository (VDB); Mayo Clinic Biobank; other disease-specific	Outpatient-based	36,000	97% European Mean age: 63 years
Mount Sinai School of Medicine	Bio*Me*^TM^, The Charles Bronfman Institute for Personalized Medicine Biobank Program	Outpatient and hospital-based	25,000	40% Hispanic/Latino 25% African 25% European
Northwestern University	NUgene	Outpatient and hospital-based	12,000	9% Hispanic/Latino 12% African 78% European Mean age: 48 years
Vanderbilt University	BioVU	Outpatient and hospital-based	155,000	2% Hispanic/Latino 15% African 80% European Mean age: 49 years

Similarly, the eMERGE consortium recently embarked upon a large-scale pharmacogenomics project [*n* = ~9000, review at Rasmussen-Torvik et al. (2012) in this issue], featuring a targeted sequencing platform developed by the Pharmacogenomics Research Network (PGRN), and covering 84 genes considered important for drug–gene interactions^4^. While the primary purpose of this project is to screen for existing pathogenic variants, this does offer an important opportunity to probe for novel variants in existing candidate genes, and to return results to patients’ medical records. This clearly cannot be accomplished without paying heed to extensive medical, psychological, and ethical considerations, which are addressed elsewhere in this issue and in previous literature (Green et al., 2013). Assuming, however, that such considerations are adequately addressed, the section below considers how this might be accomplished and the potential to impact clinical care.

## INTEGRATING CNVs WITH MEDICAL RECORDS – WHAT ARE THE OBSTACLES?

As discussed at length in this issue, the possibility of linking genomics data with EMRs represents a potentially major healthcare opportunity. What variants/results and how to report them remains open to debate, and indeed part of the remit of the eMERGE consortium is to think through these hurdles.

An obvious first step is determining the pathogenicity of relevant CNVs. Traditionally (e.g., cytogenetics), interpretation of CNVs has concentrated on diseases where the mode of inheritance was dominant, and relied on simple case–control comparisons to discriminate pathogenic from non-pathogenic variations. Where the CNV was common (i.e., frequency >1–5%), it was typically classed as non-pathogenic. Thus, by process, “rare” implied “pathogenic.” With SGS and the increased capacity to detect smaller CNVs, this assumption falls down to a certain extent. We have started to see numerous studies where control and case *de novo* rate of small CNVs is as high as 5–10%. For rare CNVs in complex diseases, there is often insufficient power on which to base a judgment. Public databases that catalog pathogenic and non-pathogenic CNVs are therefore critical to determining frequencies of CNVs in disease cases and healthy controls.

Perhaps the most widely used catalog is the DGV, which aims to provide a “comprehensive summary of structural variation in the human genome” based on peer-review of relevant studies. While the DGV has obvious clinical and research relevance, several recent commentaries (Duclos et al., 2011; Hehir-Kwa et al., 2013) have urged caution in relying too heavily on its frequency and mapping statistics. As highlighted by Lee et al. (2007), many CNVs in the DGV are derived from single platforms/technologies, which may not necessarily translate to alternate approaches. Several recent studies (Perry et al., 2008; Conrad et al., 2010) suggest that because of relatively low resolution in some studies, the size of relevant CNVs may be smaller than outlined in the DGV. Duclos et al. (2011) drew similar conclusions, stressing the “urgent need to validate the frequencies and boundaries of the CNVs recorded in the DGV.” This conclusion is based on the groups finding that some of the recorded CNVs are erroneously listed as polymorphic, which, if implemented in a medical setting may led to a deleterious CNV being called benign. Alternate CNV databases (e.g., dbVar; Lappalainen et al., 2013) have been established, but all are restrained by the quality of data on which they are based.

Other obstacles that have hampered development of CNV databases are inconsistent annotation of genomic data across studies, ill-defined curation protocols (e.g., QC-reporting, CNV-calling parameters), and incomplete phenotypic data. In each case, there is potential for consortium-led efforts to delineate best practices. To address the challenge of incomplete phenotypes, there is a particular opportunity for the eMERGE network. The majority of individuals enrolled in the eMERGE repository have their longitudinal EMRs linked to their genotype. This affords far greater potential for determining pathogenicity than traditional case–control studies, where controls may be categorized as lacking a specific disease state, with no other phenotype data. Completeness-of-EMR is critical in this regard. For patients enrolled in the biorepository at The Children’s Hospital of Philadelphia, the mean duration of EMRs is ~5.5 years, and is similar across other eMERGE sites. Relevant data include all ICD-9 diagnoses, lab values, procedures, and medications. Data of this length and depth should be considered minimal requirements for addressing pathogenicity on a large scale, while supplementation with disease-specific measures is also highly desirable.

Another major challenge in returning CNV data to patients’ EMR concerns the nature of inheritance. An interesting study by Boone et al. (2013) recently sought to determine the rate of CNVs in recessive disease genes. The group used CGH to characterize deletion CNVs in 21,470 individual, identifying 3,212 heterozygous potential carrier deletions in 419 unique disease-associated genes. While many of these CNVs are likely benign polymorphisms, the group identified 206 heterozygous CNVs in multiple recessive genes, spanning 2–6 genes in each deletion. These CNVs, therefore, confer carrier status for multiple recessive conditions. Similarly, 307 individuals had multiple deletions in recessive disease genes. While many of these gene pairs have unrelated function, a non-trivial proportion belongs to a shared pathway. Indeed, one participant had a CNV spanning three recessive immune genes *PSMB8*, *TAP1*, and *TAP2*, which are associated with autoinflammation, lipodystrophy, dermatosis syndrome (*PSMB8*), and type I bare lymphocyte syndrome (*TAP1* and *TAP2*). He also had a CNV in *CD19*, mutations of which are associated with common variable immunodeficiency. The authors were unable to determine whether the individual had a compromised immune system or presented with a history of immune disease (samples were anonymized). Nevertheless, he was clearly a multiple-deletion carrier, as were ~1.5% of the cohort: such information may be of direct clinical relevance to individuals’ offspring – whether this should be shared remains open to debate.

Inherited CNVs pose a similar set of problems. While the majority of inherited CNVs may be in loci that lead to recessive disorders, this is not always the case. Indeed, one of the best-known CNVs is duplication at 15q11–q13, which accounts for up to 3% of autism cases (Sebat et al., 2007; Marshall et al., 2008). A complex scenario was recently described by Knijnenburg et al. (2009), where a child with a homozygous deletion in 15q13.3 (inherited from non-consanguineous, hemizygous carrier parents), resulted in hearing loss. Critically, if the CNV is a gain, three copies may have no phenotypic effect but four copies may have clinical consequences (Giorda et al., 2011). Conversely, when one parent carries a CNV loss in a recessive disease gene and the other parent carries a mutation in the same gene, this can result in compound heterozygosity in offspring (Hehir-Kwa et al., 2013; Paciorkowski et al., 2013). These findings stress the point that not only is the size, location, and direction of the CNV important, but so too is the number of copies. A range of other inheritance scenarios are reviewed by Hehir-Kwa et al. (2013), including X-linked CNVs (wide vary widely across individuals), and mosaic imbalances (Kousoulidou et al., 2013; may vary across an individual’s cell types; Biesecker and Spinner, 2013; Forsberg et al., 2013).

Another point concerning CNV interpretation is the phenomenon of pleiotropy. As discussed above, a large proportion of reported recurrent CNVs have replicated *across* diseases (Cooper et al., 2011; Girirajan et al., 2011; Sahoo et al., 2011; Williams et al., 2011). Thus, the same microduplications at 1q21.1 have been associated with both autism and schizophrenia (Weiss et al., 2008; McCarthy et al., 2009). Relevant factors influencing the expressivity of this microduplication are a combination of environmental, epigenetic, and oligogenic (other modifier genes; Girirajan et al., 2010) factors. The precise mechanisms of causality that lead to a particular etiology are thus likely to be extremely complex, which calls into question what, if anything, might be reported in patients’ EMRs. Such questions are the subject of ongoing debate (Fabsitz et al., 2010; Cassa et al., 2012), and are beyond the scope of this review. However, it is obvious that as genomic data becomes increasingly ubiquitous, we will require extensive guidelines in determining how CNV results should be interpreted and shared. For the same reason, it is critical that healthcare professionals receive adequate training and resources to understand and communicate test results.

Additionally, due to large numbers of cell divisions, CNVs, particularly deletions, can be acquired in the hematogenic progenitor cells. We have previously shown that acquired mosaicism increases with age and can be associated with hematological disorders (Laurie et al., 2012; Schick et al., 2013). However, when analyzing CNVs associated with neurological disorders, such acquired CNVs must be distinguished from germline mutations that are represented in non-hematological tissues, such as brain.

## CONCLUSION

To date, a large number of diseases, across a large range of fields, have been associated with CNVs. We are still in our relative infancy in terms of deciding-upon the pathogenicity of such structural variants. We have stressed the need for a large, publicly accessible, and curated repository where CNVs that have been validated across platforms and technologies are stored. Whether this repository stems from improving existing catalogs or is developed *ab initio* remains to be determined, but the necessity of such a resource is compelling. Several eMERGE-led projects could funnel directly into such a repository, which would have real potential to impact healthcare.

A number of obstacles have stymied result-sharing – difficulties identifying CNVs (particularly in regions enriched for repetitive content), a shortage of standards, and the nature of CNV disease burden. These problems have attracted much attention in the past several years, and are well-characterized. While there is general agreement that such obstacles are substantial, there is a similar degree of optimism that benefits to be derived from solving these problems far outweigh the costs required. Again, consortium-led initiatives will likely be the most effective platforms for standardizing CNV-calling algorithms and developing guidelines for clinical care. The time is ripe for such initiatives, and we expect to see CNV-driven research make a major impact in clinical care in the next decade.

## Conflict of Interest Statement

The authors declare that the research was conducted in the absence of any commercial or financial relationships that could be construed as a potential conflict of interest.

## Footnotes

1

2

3

4
